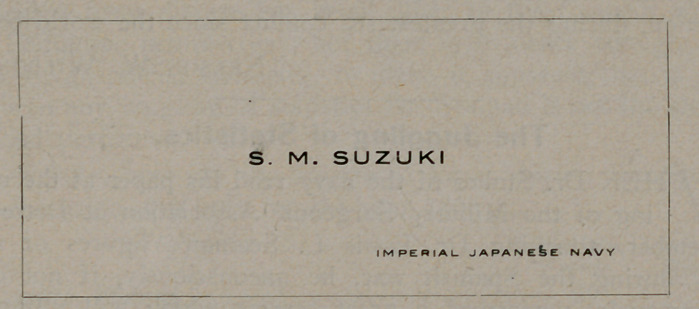# Surgeon-General Suzuki, Imperial Japanese Navy

**Published:** 1905-11

**Authors:** Nelson W. Wilson


### Surgeon-General Suzuki, Imperial Japanese Navy.

### A Pen Picture.

WITH the description of famous Japanese generals and scientists in mind; descriptions which count them as physically "little brown men," nervously active and keen-eyed, one reads of Surgeon-General Suzuki of the Japanese navy and mentally sees him a typic Nipponese. John Luther Long and many imitative writers of fictional Japanese literature are responsible for our mind pictures of the Japs; and the prolific war correspondent, held from the front by Japanese forethought, has added to the popular picture by stories in which such stilted and oft-times painfully courteous politeness is swashed about the thread of incident and made to do duty as a tale. We Americans love to be considered even-minded and cosmopolitan; we revel in the belief that we know it all and are not easily surprised; yet it must be confessed that the surgeon-general of the Imperial Japanese navy is a surprise to the Occidental mind bulging with fantastic tales of Japanese characteristics. Suzuki looks little like the Japanese we are wont to picture. He is taller and broader, being about 5 feet 8 inches in height, and while I may be committing the unpardonable sin I cannot help mentioning that, picked out of the crowd by any but a trained observer and one who has dealt extensively in the Orient, Suzuki would be taken for a Chinese gentleman, who had become Americanised to the extent of loosing his pigtail and wearing American clothing with much acquired grace and ease.

My first view of Suzuki was at the Detroit meeting of the Military Surgeons' Association at the session in which Dr. Stokes of the navy went gunning for Dr. Seaman's Japanese statistics. Suzuki sat quiet, immoveable and interested. So did the other surgeons present for that matter. Suzuki knew Seaman; he also knew the figures of the Japanese war as they were. When Seaman replied to Stokes the next morning Suzuki was an interested listener in a front seat; and when occasion required and he arose to reply to a question, I confess I expected something oratorically ornate, having once read "Madame Butterfly" and seen it Belascoed and produced as a play: which seems to meet the American idea of Japanese humility, instead of those rare tales of the cherry bloom and the chrysanthemum which were written by Lafcadio Hearn. I expected something like this:

"Your very honorable gentleman association does my poor, mean, low-down dishonorable self too much honor."

Instead of that Suzuki smiled a rather indulgent smile and spoke slowly and distinctly with just a trace of accent. "I—am afraid that---you are paying us too---high a compliment. I cannot say---anything---about the army---I was not with it.---But the navy---I was there." That was his style. Halting mayhap, feeling for proper wording possibly, yet clear, understandable and intelligent.

Coming from Detroit to Buffalo, General Suzuki's stateroom was the meeting place of several members of the association who were traveling eastward. Dr. Takamine was the general's traveling companion. They were deep in the mysteries of Nipponese converse when Dr. Seaman and I went to pay our respects to the man who stood on the bridge of Togo's flagship with that wonderful sea fighter, when he sent Rosjestvensky's fleet to the bottom of the Sea of Japan. The door of the stateroom

SURGEON-GENERAL S. M. SUZUKI.

IMPERIAL JAPANESE NAVY.

was open when we approached, yet we tapped upon it. That's the proper caper when one visits a military man. Hardly had knuckle touched wood when Suzuki jumped up with outstretched hands, his usually impassive face lighted up by a smile, a real, honest human smile, full of good nature and welcome, and not at all like the frozen-faced grin war artists and tea-pots would have us believe is the only kind of a smile they cultivate in Japan.

"Come in," he exclaimed.

Takamine, who is little and therefore typically Nipponese physically, although he is thoroughly Americanised by reason of his long residence among us, joined in the welcome. Nearly an hour's conversation followed and Suzuki was full of surprises. He bubbled over with them; yet through it all was traceable the natural reserve of the man. He told of Japanese medical work modestly and without flourish, just as if it were a matter of course; he spoke of the warm welcome he had received from the profession in this country and expressed his great gratification and pleasure; he was deeply impressed with the significance of the Military Surgeons' Association and hoped that it might eventually become an international organisation---and all this in excellent English. The only suggestion of formality was an exchange of cards.

Look at this visiting card of the Japanese navy's surgeon-general and you will get a fair idea of the simplicity of the man:

S. M. SUZUKI

IMPERIAL JAPANESE NAVY

That is all. His name and the words indicating the service to which he is attached. No one would know from this that he were other than a mere subaltern. When the visit terminated Suzuki shook hands. He gives one a firm grip and he looks one in the eye as he says:

"Good-bye---thank you for---coming. I shall see you again ---I hope---before I go."

That is the surgeon-general of the Imperial Japanese navy. A man of straightforward manner, devoid of frills and without any of the characteristics of the story-book Japanese official; a man who is much like one of us in his manner and carriage; dignified, friendly, warm-hearted and approachable, with advanced ideas, never self-opinionated. After five minutes' conversation with him one is impressed with his simplicity and his manliness, and his remarkable outward resemblance to an American, an out-and-out man with a firm grip on himself.

Look well into his face. There is purpose; purpose linked with will-power and force and character,—invaluable attributes in the make up of a military medical man. Truly, one need not wonder that the Japanese sailor man was a healthy individual during the period that Russia was getting what the fates had planned for her.

We read of the Japanese sailors changing their clothing before going into battle. That was done at Suzuki's order, so that in the event of injury the wound might be clean as maybe. One need not wonder at this remarkable exhibition of foresight when one talks with Suzuki. Togo, the bulldog of the Japanese navy, did the fighting; Suzuki kept the fighting men in fighting trim and made a military medical record which has no equal in history. And he was able to do so because he has the rank and the power to issue orders and have them obeyed without interference from a gold-laced general staff, ignorant to benightedness of sanitation or even sanitary theory.

Suzuki stands today the foremost naval surgeon in the world ---the man who saved life by preventing disease and forestalling infection, during the greatest sea conflict since the world began.

NELSON W. WILSON.

## Figures and Tables

**Figure f1:**
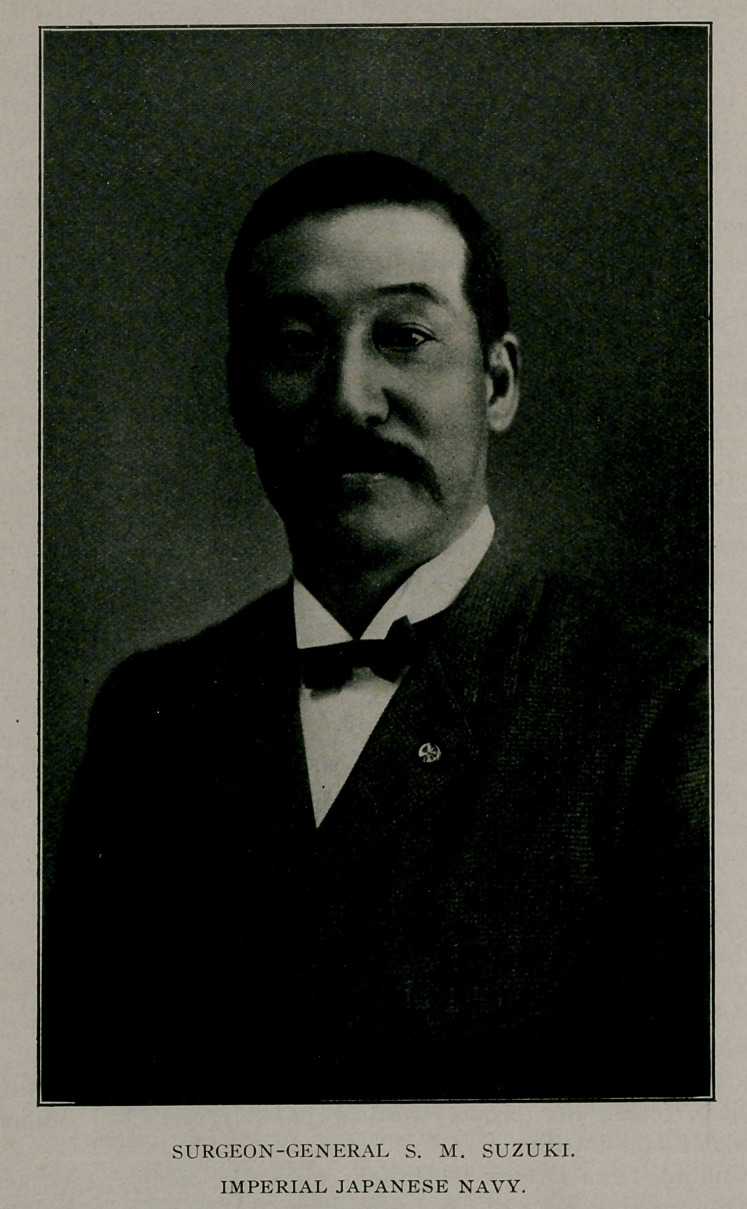


**Figure f2:**